# A Portable, Integrated, Sample-In Result-Out Nucleic Acid Diagnostic Device for Rapid and Sensitive Chikungunya Virus Detection

**DOI:** 10.3390/mi15050663

**Published:** 2024-05-19

**Authors:** Changping Xu, Yalin Chen, Guiying Zhu, Huan Wu, Qi Jiang, Rui Zhang, Beibei Yu, Lei Fang, Zhiwei Wu

**Affiliations:** 1 Center for Public Health Research, Medical School of Nanjing University, Nanjing 210093, China; 2 Zhejiang Provincial Center for Disease Control and Prevention, Hangzhou 310051, China; 3 School of Biomedical Engineering, Med-X Research Institute, Shanghai Jiao Tong University, Shanghai 200030, China; 4 Shanghai Sci-Tech InnoCenter for Infection & Immunity, Shanghai 200030, China; 5 Ustar Biotechnologies (Hangzhou) Ltd., Hangzhou 310051, China; 6 Department of Clinical Laboratory, Sir Run Run Shaw Hospital, Zhejiang University College of Medicine, Hangzhou 310000, China; 7 Department of Critical Care Medicine, Sir Run Run Shaw Hospital, Zhejiang University College of Medicine, Hangzhou 310000, China

**Keywords:** chikungunya virus, loop-mediated isothermal amplification, instrument-free nucleic acid-based detection, lateral-flow detection

## Abstract

Chikungunya virus, a mosquito-borne virus that causes epidemics, is often misdiagnosed due to symptom similarities with other arboviruses. Here, a portable and integrated nucleic acid-based diagnostic device, which combines reverse transcription-loop-mediated isothermal amplification and lateral-flow detection, was developed. The device is simple to use, precise, equipment-free, and highly sensitive, enabling rapid chikungunya virus identification. The result can be obtained by the naked eye within 40 min. The assay can effectively distinguish chikungunya virus from dengue virus, Japanese encephalitis virus, Zika virus, and yellow fever virus with high specificity and sensitivity as low as 598.46 copies mL^−1^. It has many benefits for the community screening and monitoring of chikungunya virus in resource-limited areas because of its effectiveness and simplicity. The platform has great potential for the rapid nucleic acid detection of other viruses.

## 1. Introduction

Chikungunya virus (CHIKV) is an RNA virus that belongs to the genus *Alphavirus* of the *Togaviridae* family and can cause Chikungunya fever by means of mosquito transmission [1]. It was first identified in the 1950s and has caused widespread epidemics in many parts of Asia, America, and Africa, making it as a major socioeconomic concern [2]. It is a chronic health problem with a multitude of symptoms including high fever, severe joint pain, headache, nausea, and rash [3]. More critically, infections with other arboviruses, such as dengue virus (DENV) and Zika virus, manifest with similar symptoms but are treated differently from CHIKV, which may lead to misdiagnosis. Furthermore, CHIKV can cause arthritis similar to other arthritogenic alphaviruses [4]. Therefore, accurate CHIKV diagnosis is critical for therapeutic intervention.

The gold standard for conventional CHIKV detection is virus isolation and cultivation, which takes at least 7 days and is time-consuming, labor-intensive, and unsuitable for acute infections or using in resource-limited areas [5]. The application of an enzyme-linked immunosorbent assay (ELISA) as serologic testing to detect CHIKV-specific antibodies or antigens allows for higher efficiency clinical diagnosis. However, these specific antibody/antigen-based assays show poor sensitivity in the first few days of infection, particularly during the acute phase. Clinical statistics show that anti-CHIKV lgM is only detectable from day 4 to day 7 of sickness, resulting in false-negative results [6]. Due to their higher sensitivity and specificity, molecular diagnostic techniques can detect viruses early in infection within the window period of pathogen-specific antibody/antigen detection [7]. Recently, reverse transcription-quantitative polymerase chain reaction (RT-qPCR) has been widely used in detecting various viruses, including CHIKV [8]. In recent years, progress has been made with some RT-PCR-based portable diagnosis devices, such as Visby point-of-care device for SARS-CoV-2 testing [9], but the reversion transcription and the PCR step were run separately and controlled manually with different buttons. And an external power supply is necessary. Research on an integrated RT-qPCR equipment for field detection was also published [10], but it still cannot be applied to home-based self-testing.

Isothermal nucleic acid amplification methods, such as strand displacement amplification (SDA), recombinase polymerase amplification (RPA), and loop-mediated isothermal amplification (LAMP), have also been proposed and developed [11]. Due to their simplicity, they have been utilized for virus detection in resource-restricted areas, especially RPA and LAMP. Compared to LAMP, RPA relies upon viscous crowding agents for better performance as the amplification system contains several enzymes and other protein molecules, and thus an interval mixing step after 3–6 min of incubation is recommended to distribute amplicons and improve performance [12]. LAMP, which can amplify target sequences utilizing 4–6 primers and only DNA polymerase with a strand displacement function at a constant temperature of approximately 60 °C, has been widely used for different viral diagnoses such as Zika virus [13], *Flavivirus* dengue virus [14], measles virus [15], and COVID-19 [16]. Recently, a single-tube one-step RT-LAMP assay was developed to detect CHIKV; however, it likely leads to false negatives due to the existence of amplification inhibitors, and requires an additional temperature-varying step for characterization [17]. Other LAMP amplicon detection approaches, such as turbidity measurement, fluorescence dye hybridization, electrochemical luminescence, melting curves analysis, etc., require expensive equipment for fluorescence detection, or additional detection such as melting curves analysis, which limits their application in clinical diagnosis, especially in resource-limited areas [18,19]. Thus, there is a vital need for an integrated platform for CHIKV identification, which includes sample treatment and specific CHIKV target sequence amplification.

Herein, a portable device named AutoLAMP, which integrates RT-LAMP amplification and lateral-flow detection (LFD), has been developed for the diagnosis of CHIKV following a few simple steps. The CHIKV RNA in serum samples can be dispersed and amplified in specific wells of the device via microchannels for 35 min. Subsequently, the amplicon can be detected in the LFD module and the output result is visible from the result window. The whole process requires no professional operation or additional equipment. The device has high sensitivity and specificity, demonstrating great potential for application in community and mass screening in undeveloped areas.

## 2. Materials and Methods

### 2.1. Clinical Specimens, Virus Isolates, and Viral RNA Extraction

Serum samples were collected from suspected cases of Chikungunya fever monitored between 2012 and 2019 in Zhejiang province, China. A modified method based on the classic cDNA-AFLP and sequence analyses was used to identify the probable virus isolate. A suspected virus was finally identified as CHIKV, with the virus strain named chik-sy/2012 (GenBank accession number: KF318729) [20]. In addition, *Flavivirus* dengue virus (DENV) type 1–4, Zika virus, *Flavivirus* Japanese encephalitis virus, and yellow fever virus were selected for specificity verification.

Viral RNA was extracted using an RNeasy Mini Kit (Qiagen, Hilden, Germany) according to the manufacturer’s instructions. The extracted RNA was stored at −80 °C for subsequent analysis.

### 2.2. Preparation of CHIKV RNA Standards

Synthetic RNA was also generated from chik-sy/2012. Firstly, the nonstructural polyprotein gene sequence of the chik-sy/2012 strain was amplified with primers that included the T7 promoter sequence. The forward and reverse primer sequences were 5′-ACTCGTTAATACGACTCACTATAGGGAG-CTCTCCTCTCCACAGGTGTA-3′ (T7 promoter sequence is the former part) and 5′-CGCAGTCTATGGAGATGTGC-3′, respectively. The product was in vitro transcribed with T7 RNA polymerase (TaKaRa Biotechnology Co., Otsu, Japan) according to the manufacturer’s instructions. Finally, the synthetic RNA transcript was purified, quantified, and diluted.

### 2.3. RT-LAMP Reaction and Analysis of RT-LAMP Amplicons

Different genome sequences of various CHIKV isolates (including the West African, East/Central/South African, and Asian lineages) were downloaded from GenBank. Multiple sequence alignments were performed using DNAMAN version 6.0 software. The conserved regions of the nonstructural polyprotein sequences were utilized for LAMP primer design by using PrimerExplorer Version 4 (http://primerexplorer.jp/elamp4.0.0/index.html, accessed on 1 May 2019; Eiken Chemical Co., Tokyo, Japan). Loop primers were designed manually, with the 5′ ends of LF and LB labeled with FITC and biotin, respectively. The designed primer sequences were shown in Table 1. Primers were tested for hybrids and hairpin structures using the Integrated DNA Technologies design tools (http://eu.idtdna.com/pages/scitools, accessed on 9 May 2019). All of the oligoes were synthesized by TaKaRa Biotechnology Co., Ltd. (Dalian, China).

RT-LAMP reactions were performed in 50 μL reaction mixtures containing 0.8 μM of FIP and BIP, 0.4 μM of LF and LB, 0.2 μM of the outer primers F3 and B3, 1.4 mM of each deoxynucleoside triphosphate (dNTP), 8 mM MgSO_4_, 0.2 M betaine, 50 mM Tris-HCl (pH 8.1), 30 mM KCl, 30 mM (NH_4_)_2_SO_4_, 1% Triton X-100, 8 U of avian myeloblastosis virus reverse transcriptase (Bioer Technology Co., Ltd., Hangzhou, China), 10 U of Bst DNA polymerase large fragment (NEB, Beverly, MA, USA), and 20 μL of target RNA. The RT-LAMP reactions were incubated at 60 °C for 40 min, and the real-time amplification and detection process was monitored using a Loopamp Real-time Turbidimeter (LA-500; Eiken Chemical Co., Ltd., Tochigi, Japan). After the reaction, 5 μL of RT-LAMP products were electrophoresed on 1.5% agarose gel and the results were observed under an ultraviolet imager. 

### 2.4. Optimization of RT-LAMP

Temperatures ranging from 54 to 64 °C (with an interval of 2 °C) were examined to determine the optimal temperature using the RT-LAMP reaction system mentioned above. The RT-LAMP amplicons were detected using lateral-flow test strips. The synthetic CHIKV RNA at concentrations of 2 × 10^5^, 2 × 10^4^, 2 × 10^3^, 2 × 10^2^, and 2 × 10^1^ copies mL^−1^ were tested.

Reaction times ranging from 20 to 40 min (with an interval of 5 min) were examined to determine the optimal RT-LAMP reaction time in a similar manner.

### 2.5. RT-qPCR Assay

RT-qPCR was performed using the One Step PrimeScript RT-qPCR (Perfect Real-time) Kit (TaKaRa Biotechnology Co., Dalian, China). The forward and reverse primer sequences were 5′-CAT CTG CAC YCA AGT GTA CCA-3′ and 5′-GCG CAT TTT GCC TTC GTA ATG-3′. The probe sequence was 5′ FAM-GCG GTG TAC ACT GCC TGT GAC YGC-BHQ1 3′. Each 50 μL reaction mixture contained 25 μL of 2 × One-Step RT-QPCR Buffer, 1 μL of TaKaRa Ex Taq™ HS, 1 μL of PrimeScript™ RT Enzyme Mix, 0.48 µM each primer, 0.24 µM probe, and 20 μL of RNA template. The amplification conditions were as follows: 30 min at 42 °C for reverse transcription, 2 min at 95 °C for the activation of the Taq HS enzyme, and 40 cycles of amplification at 94 °C for 10 s and 55 °C for 35 s. The fluorescence signal was obtained at the end of each annealing step.

### 2.6. AutoLAMP-Based CHIKV Detection

For AutoLAMP-based CHIKV detection, approximately 50 μL of sample after lysis with rapid lysis buffer (Ustar Biotechnologies, Hangzhou, China) was introduced to the sample hole, followed by the addition of about 100 μL of buffer A (Ustar Biotechnologies, China) to cause the sample liquid to flow into the amplification room through a microchannel. The presence of the sample liquid dissolved the embedded RT-LAMP reaction components in the silica membrane which was set in the amplification room through the microchannel. Then, the amplification room was heated to 58 ± 2 °C for approximately 35 min for the RT-LAMP reaction using the underlying heating module with a built-in battery and chip. After amplification, about 100 μL of buffer A was added into the sample hole, allowing the LAMP amplicon to flow into the microchannel and to be detected by the test strip in the LFD module in 3–5 min. A positive result produced two red bands on both the test and control lines, whereas a negative result created only one band on the control line. For test validation, a clearly visible red-purple band must appear at the control line.

### 2.7. Sensitivity and Specificity

The specificity of AutoLAMP-based CHIKV detection was identified by analyzing the genomic nucleic acid of CHIKV, *Flavivirus* dengue viruses with four serotypes, *Flavivirus* Japanese encephalitis virus, Zika virus, and yellow fever virus.

The sensitivity of the AutoLAMP and real-time RT-qPCR for CHIKV detection was determined by testing serial 10-fold dilutions of virus RNA (2 × 10^5^, 2 × 10^4^, 2 × 10^3^, 2 × 10^2^, and 2 × 10^1^ copies mL^−1^). The lowest detectable concentration was compared with the sensitivity of RT-qPCR.

The analytical sensitivity was determined using CHIKV virions quantified by digital droplet PCR (ddPCR). The virions was diluted with uninfected serum to obtain two-fold dilutions. Rapid lysis buffer was used to extract RNA from all samples. The limit of detection (LOD) was determined through the amplification of the serial two-fold dilution of serum samples in twenty replicates. The LOD was defined as the concentrations (unit: copies/mL) of the lowest dilution at which all replicates could be detected with at least 95% probability.

## 3. Results 

### 3.1. Illustration of AutoLAMP-Based RT-LAMP-LFD CHIKV Detection

**Figure 1:**
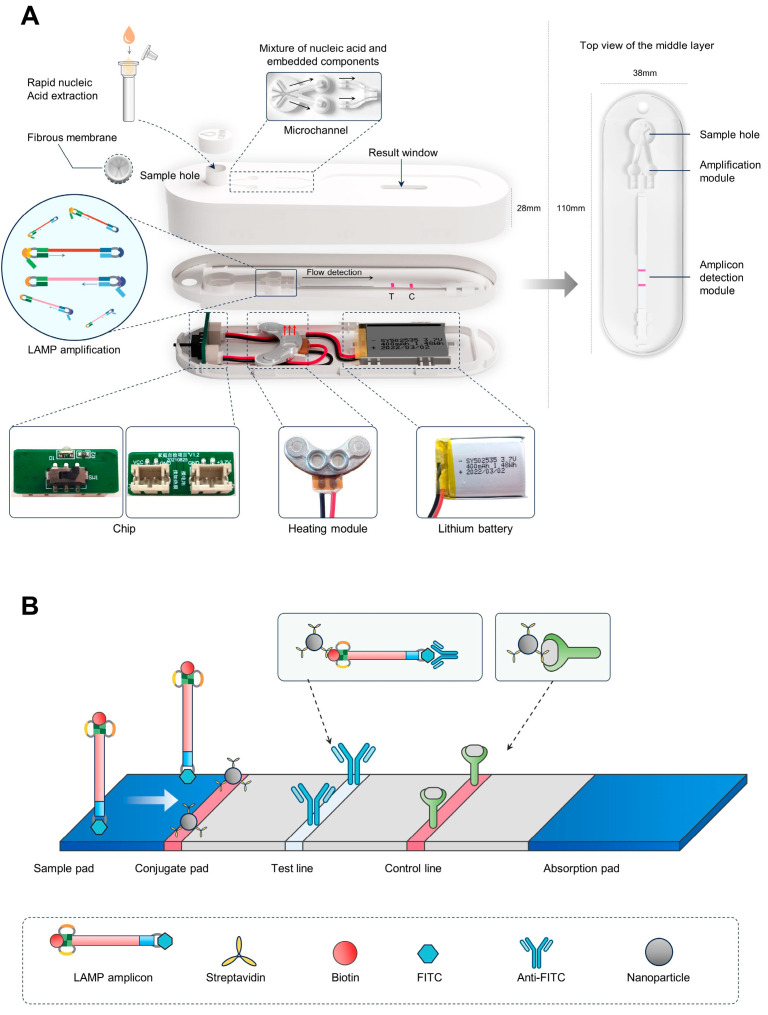
Illustration of the AutoLAMP-based rapid CHIKV detection. (**A**) The principle and structure of AutoLAMP. (**B**) The principle of the lateral-flow detection in AutoLAMP.

The AutoLAMP device combined RT-LAMP amplification and LFD for the identification of CHIKV, with naked-eye-readable results (Figure 1). The cassette mainly contains three parts: a sample hole, the amplification module, and the amplicon detection module. The sample hole blocks solid impurities in the sample through the upper fibrous membrane. The amplification module includes reaction rooms with a heater which provides a closed compartment for RT-LAMP under a constant temperature and reduces liquid evaporation. The heater integrates a DC 3.7V lithium battery for power supply, a heating module for heat conduction, and a circuit chip set heating program. The heating temperature is stable in the range of 2–30 °C ambient temperature, ensuring the optimal performance of the amplification. The LFD test strip in the amplicon detection module enables the quick lateral-flow testing of the RT-LAMP products and visual results from the result window. The three parts are connected sequentially through the microchannels, and the sample is processed step-by-step for reactant dissolution with sample liquid, amplification, and detection. The microchannels also provide a fully enclosed environment for reactant preparation, amplification, and amplicon detection, which prevents aerosol contamination and avoids false positive results.

The workflow of the AutoLAMP is shown in Figure 1. The serum sample is lysed by the rapid lysis buffer and introduced into the cassette from the sample hole, where a fibrous membrane blocks impurities in the specimen (Figure 1A). The lyophilized reactants adsorbed on the silica membrane are dissolved and mixed at the addition of the loading buffer, which enables the extracted nucleic acid to flow into the reaction room via the microchannel. Then, the LAMP reaction starts at 58 ± 2 °C set by the built-in battery and circuit chip in the heater. After amplification, the amplicon flows into the microchannels at the addition of the loading buffer, and is detected by the LFD strip. The LFD strip contains an anti-FITC antibody in the test line and streptavidin-coated gold nanoparticles in the sample pad (Figure 1B). 

### 3.2. RT-LAMP Using LFD for CHIKV Detection

The genome sequences of various CHIKV isolates (including the West African lineage, the East Central South African lineage, and the Asian lineage) were obtained from GenBank. Based on sequence alignments, LAMP primers were designed at the conserved portions of different strains (Table 1). By designing degenerate nucleotides at individual single-base difference sites, it was intended that the primers have adequate coverage for the various CHIKV lineages.

**Figure 2:**
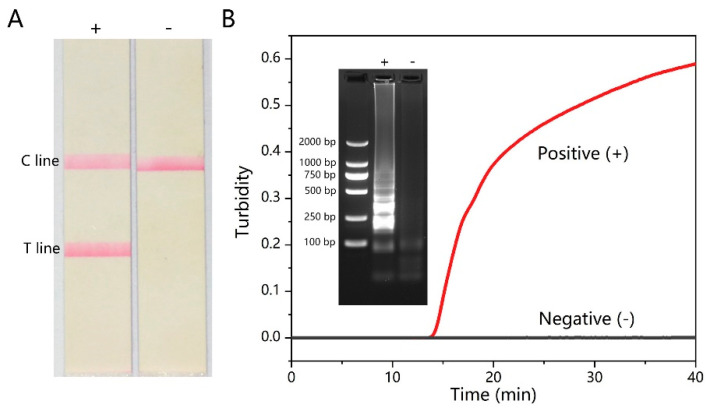
Validation of detecting CHIKV with RT-LAMP. (**A**) LFD results of RT-LAMP. (**B**) Turbidity–time curve and gel agarose electrophoresis of RT-LAMP.

The amplicon of the RT-LAMP amplification of CHIKV was validated using a variety of methods, including LFD, agarose gel, and a turbidimeter. The results of LFD are shown in Figure 2A; only the reaction with the presence of the CHIKV genome resulted in the positive readout. The positive RT-LAMP reaction displayed bands of different sizes in agarose gel electrophoresis, whereas the negative samples showed no band, which was consistent with the LFD results (Figure 2B). The real-time amplification process of RT-LAMP can also be monitored using the turbidimeter. The turbidity of the positive reaction only increased when the incubation time increased (Figure 2B). 

### 3.3. Optimization of RT-LAMP-LFD for the AutoLAMP

**Figure 3:**
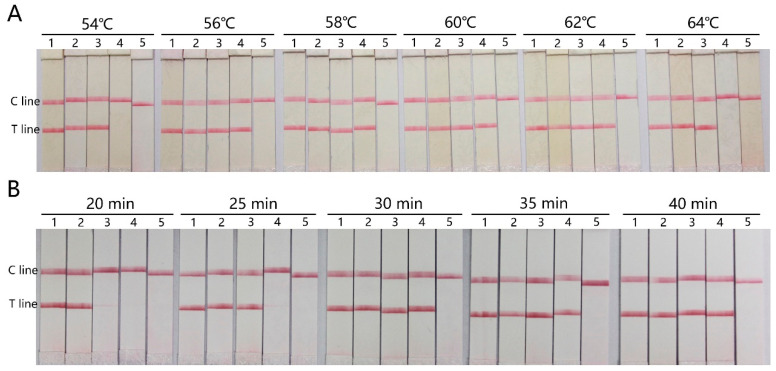
Optimization of the reaction conditions of RT-LAMP based on LFD. (**A**) Optimization of the RT-LAMP reaction temperature: (1) 2 × 10^5^, (2) 2 × 10^4^, (3) 2 × 10^3^, (4) 2 × 10^2^, and (5) 2 × 10^1^ copies mL^−1^. (**B**) Optimization of the RT-LAMP reaction time: (1) 2 × 10^5^, (2) 2 × 10^4^, (3) 2 × 10^3^, (4) 2 × 10^2^, and (5) 2 × 10^1^ copies mL^−1^.

The conditions of the RT-LAMP reaction based on LFD were evaluated and optimized (Figure 3). Different reaction temperatures and incubation times were tested to obtain a higher sensitivity. Since the temperature given by the heating module within AutoLAMP varies between 56 and 60 °C, reaction temperatures ranging from 54 to 64 °C were selected with target concentrations from 2 × 10^5^ copies mL^−1^ to 2 × 10^1^ copies mL^−1^. The LFD results showed that the detectable concentration was down to 2 × 10^2^ copies mL^−1^, which was not affected by temperatures from 56 to 62 °C (Figure 3A). The optimal reaction time was also evaluated in the same range of the target concentration. LFD strips with the target concentration of 2 × 10^2^ copies mL^−1^ showed an increase in signal intensity with longer incubation time from 20 to 40 min, and the positive results were maintained from 30 to 40 min (Figure 3B). LFD strips with the target concentration of 2 × 10^1^ copies mL^−1^ were undetectable in all conditions. Thus, the temperature given by the heating module (58 ± 2 °C) within AutoLAMP could generate the highest RT-LAMP sensitivity, and 35 min is prescribed as the optimal reaction time. 

### 3.4. Specificity and Sensitivity of AutoLAMP-Based CHIKV Detection

**Figure 4:**
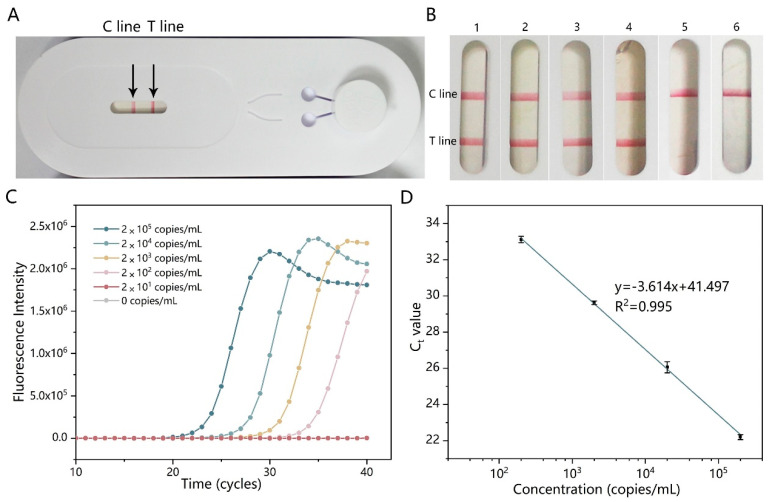
Sensitivity of the AutoLAMP-based RT-LAMP-LFD using artificial CHIKV virus. (**A**) Full-view demonstration of the AutoLAMP device using for CHIKV detection. (**B**) Test strip results of RNA based on AutoLAMP with different concentrations (enlarged view): (1) 2 × 10^5^, (2) 2 × 10^4^, (3) 2 × 10^3^, (4) 2 × 10^2^, (5) 2 × 10^1^, and (6) 0 copies mL^−1^. (**C**) The corresponding RT-qPCR results of the concentration-gradient synthetic RNA. (**D**) Regression curve of the Ct value of RT-qPCR.

The specificity of the AutoLAMP-based CHIKV detection was demonstrated using the extracted genomic nucleic acid of other arboviruses that exhibited similar symptoms, including *Flavivirus* dengue viruses of four serotypes, *Flavivirus* Japanese encephalitis virus, Zika virus, and yellow fever virus. Only the test strip using CHIKV RNA as the template obtained a positive result, indicating that the assay has no cross-reactivity with other viruses. The positive result can be read directly on the device, as shown in Figure 4A.

The sensitivity of our assay was evaluated using 10-fold serially diluted viral RNA, from 2 × 10^5^ to 2 × 10^1^ copies mL^−1^. As shown in Figure 4B, the sensitivity of the LFD detection was 2 × 10^2^ copies mL^−1^. The sensitivity of AutoLAMP was also compared to that of RT-qPCR, and the results indicated that the two methods had a similar sensitivity around 2 × 10^2^ copies mL^−1^ (Figure 4C,D).

### 3.5. Evaluation of AutoLAMP Assay with Clinical Samples

**Figure 5:**
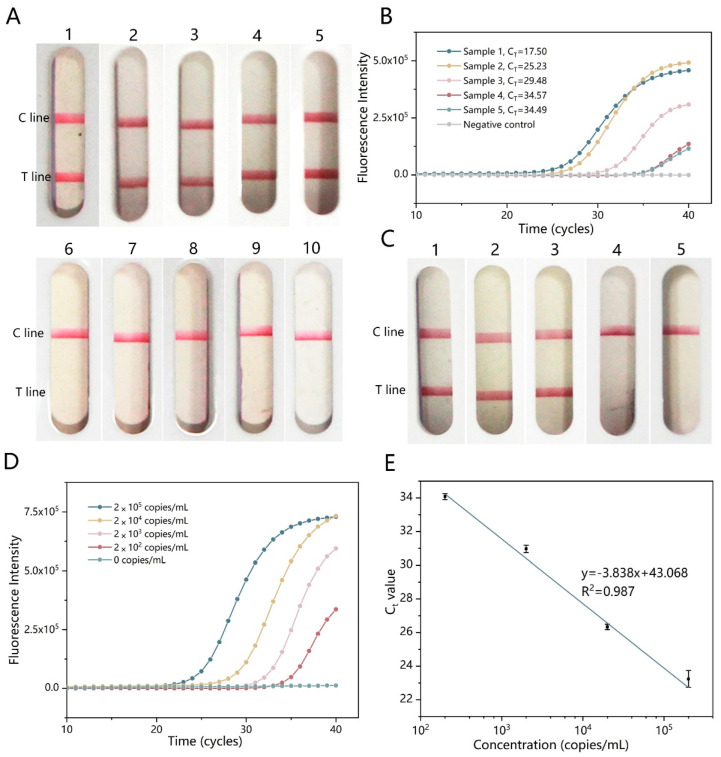
Performance of AutoLAMP with clinical samples. (**A**) Representative AutoLAMP results of serum samples using fast lysis buffer (enlarged view): (1) to (5) CHIKV, (6) dengue virus type 1, (7) dengue virus type 2, (8) Zika virus, and (9) and (10) healthy donors. (**B**) Results of five clinical CHIKV samples amplified with RT-qPCR. (**C**) AutoLAMP results using 10 times serial diluted serum samples (enlarged view): (1) 2 × 10^5^, (2) 2 × 10^4^, (3) 2 × 10^3^, (4) 2 × 10^2^, and (5) 0 copies mL^−1^. (**D**) Results of CHIKV virion samples at serial concentrations and with RT-qPCR. (**E**) Regression curve of the Ct value of RT-qPCR.

The clinical CHIKV samples from suspected patients were assayed to further validate the performance of the AutoLAMP device. A total of 10 serum samples, comprising 5 CHIKV infected serum samples and 5 other serum samples, were collected and analyzed as shown in Figure 5A. Positive results were only obtained in the CHIKV serum samples with no cross-reaction. The CHIKV-infected serum samples were also detected utilizing RT-qPCR (Figure 5B). Compared with the results of AutoLAMP and RT-qPCR, the virus samples with a low content displayed a similar shade of the color on the strips to that of samples with a high content, suggesting the good amplification capability of AutoLAMP for the samples with a low virus load within the detectable range.

In order to simulate various virus concentrations in clinical samples, CHIKV virions (2 × 10^6^ copies mL^−1^) were diluted 10 times with serum from healthy individuals. The detection results of these samples, which were pretreated with rapid lysis buffer and processed with AutoLAMP, were compared with the results of viral RNA extracted using a commercial RNeasy Mini Kit (Qiagen, Germany), and run using RT-qPCR. The test strip yielded positive results when the concentration was above 2 × 10^3^ copies mL^−1^ (Figure 5C). These results indicated that the sensitivity of samples treated with rapid lysis buffer followed by AutoLAMP is roughly 10 times lower than that of the virus detected with commercial extraction kits and the RT-qPCR process (Figure 5D,E). However, we demonstrated that the sensitivity of AutoLAMP was the same as that of RT-qPCR when detecting the synthetic viral RNA, indicating that sample pretreatment using rapid lysis buffer may affect the sensitivity of AutoLAMP. 

Further, the limit of detection (LOD) of the AutoLAMP was investigated using a two-times series diluted virus ranging from 2000 to 125 copies mL^−1^; all samples were pretreated using rapid lysis buffer. All 20 tests were positive with concentrations over 1000 copies mL^−1^, while 16 of 20 and 8 of 20 were positive with concentrations of 500 and 250 copies mL^−1^, respectively (Table 2). However, there were no positive results when the target was down to 125 copies mL^−1^. Thus, the LOD of the AutoLAMP device was determined to be 598.46 copies mL^−1^ with 95% CI.

## 4. Discussion

CHIKV is a mosquito-borne RNA virus that is primarily found in regions of developing countries, such as Africa, Asia, and the Americas [21]. Because the symptoms of its infection are similar to other arboviruses, it often leads to misdiagnosis, which delays proper treatment. Conventional virus detection methods, including virus isolation, anti-CHIKV lgM detection, and RT-qPCR, have their respective limitations, such as being time-consuming and having a narrow detection window period and high laboratory requirements.

In this study, we developed an AutoLAMP point-of-care platform for CHIKV rapid diagnosis by integrating RT-LAMP and LFD. Its optimal sensitivity reached 200 copies mL^−1^ when evaluated by 10 times diluted purified RNA templates, similar to RT-qPCR. The LOD of AutoLAMP was 598.46 copies mL^−1^ with further quantification using virion samples. Meanwhile, it had a high specificity and could effectively differentiate CHIKV from dengue virus, Zika virus, Japanese encephalitis virus, and yellow fever virus. Moreover, the results demonstrated that our device could be used for the analysis of clinical samples with high efficiency.

The present work still has some limitations. The practical application in a clinical setting needs further validation with a larger number of clinical samples. Since China is not the indigenous epidemic region of chikungunya fever and has no local cases, the method will need to be tested and validated in epidemic regions through collaborative efforts. The specificity, sensitivity, and reproducibility of the AutoLAMP device-based assay should be further assessed with a large number of clinical samples. Moreover, the platform can be further improved by integrating the process of sample collection and pretreatment.

Since the AutoLAMP device-based platform can complete the integrated process of reactant mixture, amplification, and detection in a relatively closed environment, it can prevent cross contamination and reduce false positives. Moreover, due to the simple operation, precise equipment-free nature, and high sensitivity of the AutoLAMP device, it can also be used for the detection of other viruses, such as *Ebolavirus*, with high efficiency at community hospitals or resource-limited areas.

## Figures and Tables

**Table 1:** Primers used for the rapid CHIKV detection of AutoLAMP-based RT-LAMP-LFD.

Primer Name	Sequences (5′ to 3′)
CHIKV-F3	CTCTCCTCTCCACAGGTGTA
CHIKV-B3	CGCAGTCTATGGAGATGTGC
CHIKV-FIP(F1c + F2)	CCGTCGAGTCCATR^a^ GCTGTAAA- ACTCAGGARGGAAAGACAGG
CHIKV-BIP (B1c + B2)	GCAGACGTGGTCATCTACTGCC- ACTTGRGTCCGCATCTGT
CHIKV-LF	FITC-TGGTTCAGTGACTGGGTYAG
CHIKV-LB	Biotin-ATGGGAGAAGAARATATCYGAGGC

^a^ The individual degenerate nucleotides are underlined.

**Table 2:** Limits of detection of the AutoLAMP-based RT-LAMP-LFD for the detection of CHIKV.

RNA Concentration (Copies mL^−1^)	No. Positive Tests/No. Reaction Replicates (%)	95% Cl
2000	20/20 (100%)	83.90–100%
1000	20/20 (100%)	83.90–100%
500	16/20 (80%)	58.40–91.93%
250	8/20 (40%)	21.88–61.34%
125	0/20 (0%)	0.00–16.11%

## Data Availability

The original contributions presented in the study are included in the article, further inquiries can be directed to the corresponding author.
